# Non-destructive 3D imaging method using muonic X-rays and a CdTe double-sided strip detector

**DOI:** 10.1038/s41598-022-09137-5

**Published:** 2022-03-28

**Authors:** I-Huan Chiu, Shin’ichiro Takeda, Meito Kajino, Atsushi Shinohara, Miho Katsuragawa, Shunsaku Nagasawa, Ryota Tomaru, Goro Yabu, Tadayuki Takahashi, Shin Watanabe, Soshi Takeshita, Yasuhiro Miyake, Kazuhiko Ninomiya

**Affiliations:** 1 Radioisotope Research Center, Institute for Radiation Sciences, Osaka University, 1-1, Machikaneyama, Toyonaka Osaka, 560-0043 Japan; 2 Kavli Institute for the Physics and Mathematics of the Universe (WPI), The University of Tokyo, 5-1-5 Kashiwanoha, Kashiwa Chiba, 277-8583 Japan; 3 Graduate School of Science, Osaka University, 1-1, Machikaneyama, Toyonaka Osaka, 560-0043 Japan; 4 Department of Physics, The University of Tokyo, 7-3-1 Hongo Bunkyo, Tokyo, 113-0033 Japan; 5 Present Address: Institute of Space and Astronautical Science, Japan Aerospace Exploration Agency (ISAS/JAXA), 3-1-1 Yoshinodai, Chuo-ku, Sagamihara Kanagawa, 252-5210 Japan; 6 High Energy Accelerator Research Organization (KEK), Tsukuba, Ibaraki, 305-0801 Japan; 7 Faculty of Health Science, Osaka Aoyama University, 2-11-1 Niina, Minoh, Osaka, 562-8580 Japan

**Keywords:** Imaging techniques, Physics, Exotic atoms and molecules

## Abstract

Elemental analysis based on muonic X-rays resulting from muon irradiation provides information about bulk material composition without causing damage, which is essential in the case of precious or otherwise unreachable samples, such as in archeology and planetary science. We developed a three-dimensional (3D) elemental analysis technique by combining the elemental analysis method based on negative muons with an imaging cadmium telluride double-sided strip detector (CdTe-DSD) designed for the hard X-ray and soft $\gamma$-ray observation. A muon irradiation experiment using spherical plastic samples was conducted at the Japan Proton Accelerator Research Complex (J-PARC); a set of projection images was taken by the CdTe-DSD, equipped with a pinhole collimator, for different sample rotation angles. The projection images measured by the CdTe-DSD were utilized to obtain a 3D volumetric phantom by using the maximum likelihood expectation maximization algorithm. The reconstructed phantom successfully revealed the 3D distribution of carbon in the bulk samples and the stopping depth of the muons. This result demonstrated the feasibility of the proposed non-destructive 3D elemental analysis method for bulk material analysis based on muonic X-rays.

## Introduction

Elemental analysis plays an important role in various scientific fields. At present, several analytical methods, including optical emission spectrometry and mass spectrometry, are currently used for the high-quality and reliable characterization of various samples. Among them, methods that do not cause damage to the sample are strongly desired for the analysis of valuable materials and objects. One such non-destructive analysis method, X-ray fluorescence spectroscopy^1^, is widely used in various fields such as archaeology and planetary science; however, it can only provide the elemental composition only for the near-surface of the sample and is not suitable for accurate quantification, especially as regards light elements. In the last decade, a non-destructive elemental analysis method using negatively charged muons has been developed^2^. When a negative muon is captured in the irradiated material, a “muonic atom” is formed. Since the mass of a muon is 207 times that of an electron, the characteristic muonic X-rays emitted from the as-formed muonic atoms have high energy due to the binding energy of a muon in a muon atomic orbital; therefore, except for muonic hydrogen atoms that emit low-energy X-rays, muonic X-rays from any element can be detected with high sensitivity without being absorbed by the sample itself^3^. Due to the unique characteristics of the muonic X-rays, the proposed method has been used to measure the concentrations of light elements in bulk materials, such as carbon in the interior of meteorites^4,5^. By adjusting the energy of the incoming muons that are accelerated by a high-energy accelerator, the stopping position of the muons within the sample material can be easily controlled. Elemental depth profiling of archeological samples can also be achieved^6,7^.

Germanium and silicon detectors have been commonly used for hard X-ray measurements in elemental analyses because of their high-energy resolution. However, the low operating temperature of the formers due to their small bandgap and the low stopping power of the latter limit their applications. Cadmium telluride semiconductor detectors, where cadmium and tellurium both have large atomic numbers (48 and 52, respectively), provide high sensitivity for X-ray measurements due to their high photon detection efficiency^8^. An elemental imaging experiment based on muonic X-rays measurement using the pixelated CdTe detector has been discussed by Hiller et al.^9^. Besides, Takahashi et al.^10,11^ reported the stability performance of the Schottky CdTe diodes at an operating temperature of $5^\circ$. A large-area CdTe double-sided strip detector (CdTe-DSD) has been recently developed for two-dimensional (2D) imaging analysis for hard X-ray measurements^12,13^. Due to its high spatial resolution, the CdTe-DSD is the best choice for imaging based on negative muonic X-rays. Katsuragawa et al. performed the 2D visualization of light elements, i.e., boron and nitrogen, in the standard samples by using muonic X-rays and the CdTe-DSD^14^. By adjusting the muon beam energy, muonic X-ray images with depth profiling for the standard samples have also been achieved in this study; however, the depth profiling cannot be accurately measured for a real sample due to its unknown elemental distribution. In addition, the momentum spread of the muon beam also affects the stopping depth of the muon. Therefore, a method to generate an advanced three-dimensional (3D) visualization based on the computed tomography (CT) with high-accuracy position estimation must still be developed. The reconstruction of 3D volumetric phantom would hugely benefit the elemental composition analysis. In the present study, we designed an experiment using our knowledge of the CT technology and an imaging system to accomplish such as 3D phantom reconstruction. The imaging system, fabricated by Katsuragawa et al.^14^, consists of the 3 mm diameter tungsten-made pinhole collimator and the CdTe-DSD, which has a spatial resolution of 250 $\upmu$m and a 32 mm-wide solid CdTe crystal with a thickness of 750 $\upmu$m. This is the first result with tomographic images based on 3D elemental analysis method using muon beam.

We experimentally assessed the feasibility of 3D imaging via muon elemental analysis coupled with an imaging CdTe-DSD detector. As the samples, we prepared four spherical plastic balls, which were rotated with a step size of $22.5^\circ$ each time during muon irradiation. 16 independent projection images recorded by the CdTe-DSD were used with the maximum likelihood expectation maximization (MLEM) algorithm^15^ to reconstruct a 3D phantom of the sample shape.

## Results and discussion

### Non-destructive elemental analysis

**Figure 1:**
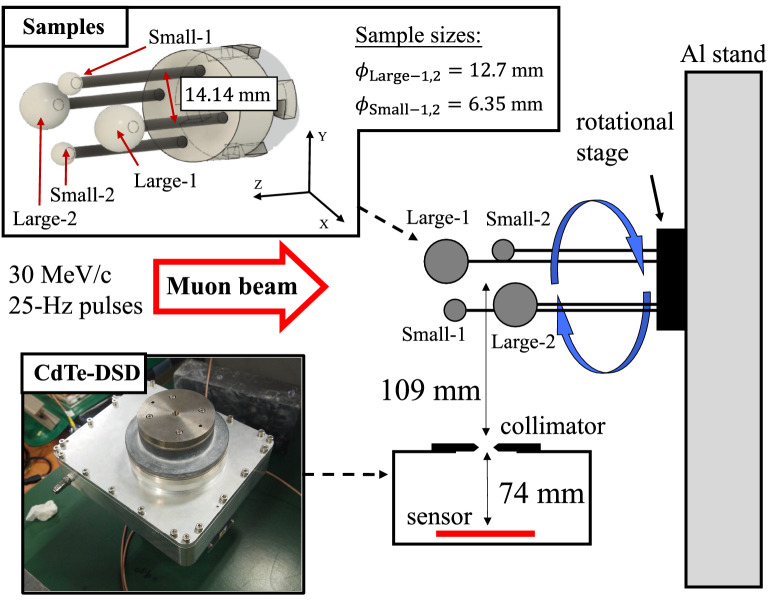
Geometry of the imaging experiment.

Figure 1 illustrates the experimental setup. The samples were four polypropylene (PP) balls, two larger ones with a diameter of 12.7 mm (Large-1 and Large-2) and two smaller ones with a diameter of 6.35 mm (Small-1 and Small-2). They were fixed at the aluminum columnar stand in air, at the center of the system, and irradiated with the negative muon beam. As Fig. 1, the CdTe-DSD was installed at the bottom of the system, and the distances from the pinhole collimator to the sample and from the pinhole collimator to the CdTe sensor were 109 and 74 mm, respectively. The samples were rotated $22.5^\circ$ around the muon beam axis every 30 min during the muon irradiation. This rotation enabled the CdTe-DSD to acquire projection images of the samples at various angles over the $0^\circ$–$360^\circ$ range. The exposure to the muon beam with a momentum of 30 $\mathrm {MeV}/c$ lasted about 34 h; in this beam profile, the number of negative muons was in the order of $10^{6}$ per second. We used Geant4^16^, a toolkit for Monte Carlo simulations, to verify the signal and background sources. Due to the molecular structure of PP $(\mathrm {C}_3\mathrm {H}_6)_n$, the signals detected by the CdTe-DSD were confirmed to be the muonic X-rays from hydrogen and carbon ($\upmu$H and $\upmu$C, respectively). Moreover, the muonic X-rays from the Al columnar stand ($\upmu$Al) represented a considerable background noise.

### Spectral analysis

**Figure 2:**
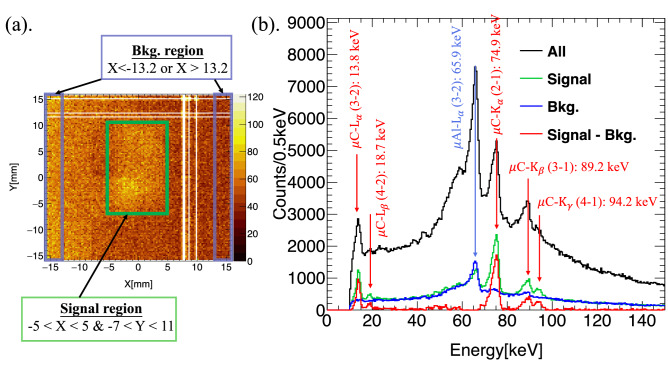
(**a**) Hit position of the incident photons with energy larger than 10 keV on the CdTe-DSD. The white bands in the image are the non-functioning strips. (**b**) Energy spectra of the signal and background regions.

The readout signals on the anode and cathode sides of the CdTe-DSD are associated with the energy deposits of hard X-rays or $\gamma$-rays in the CdTe semiconductor. They were identified as $E_{\mathrm {anode}}$ and $E_{\mathrm {cathode}}$, respectively, and used to determine the energy of the incident photon. First, in consideration of the electronic noise effect on the device, an event was discarded if $E_{\mathrm {anode}}$ or $E_{\mathrm {cathode}}$ was lower than 10 keV; then, the energy of the incident photon was derived by averaging $E_{\mathrm {anode}}$ and $E_{\mathrm {cathode}}$. To better identify the muonic X-rays from the samples, the signal and background regions were defined based on the hit positions of the incident photons on the CdTe sensor, as illustrated in Fig. 2a. For quantitative comparison, the two regions had the same total area and their corresponding energy spectra are shown in Fig. 2b, which also displays the spectrum of all the photons in the energy range from 0 to 150 keV and the one obtained subtracting the background noise to the signal spectrum. These spectra demonstrate that the CdTe-DSD clearly detected the signal from the samples, which was characterized by five peaks with the weighted arithmetic means^17^ of 13.8, 18.7, 74.9, 89.2, and 94.2 keV. According to Engfer et al.^18^, all the peaks were $\upmu$C from the samples that; however, were made on carbon and hydrogen. Although $\upmu$H was one of the signals to consider in this experiment, the CdTe-DSD could detect only $\upmu$C since the energy of $\upmu$H ($\sim$2 keV) was outside the detectable energy range. Among the detected peaks, $\upmu$C $K_{\alpha }$ (muonic Lyman X-rays, 74.9 keV) and $\upmu$C $L_{\alpha }$ (muonic Balmer X-rays, 13.8 keV) had the strongest intensity. The background spectrum exhibited a clear peak at 65.9 keV and a continuum component associated with the Compton effect and bremsstrahlung emission. Based on the Monte Carlo simulation, we confirmed that the background noise represented the muonic X-rays from the Al columnar stand ($\upmu$Al $L_{\alpha }$), whose energy is 65.9 keV. Since the total cross-sectional area of the samples was much smaller than the diameter of the muon beam, significant fraction of muons passed the sample and stopped behind the Al columnar stand, which was installed downstream of the muon beam. Thus, $\upmu$Al $L_{\alpha }$ from the Al columnar stand was the main background contribution in this experiment.

### Projection images

**Figure 3:**
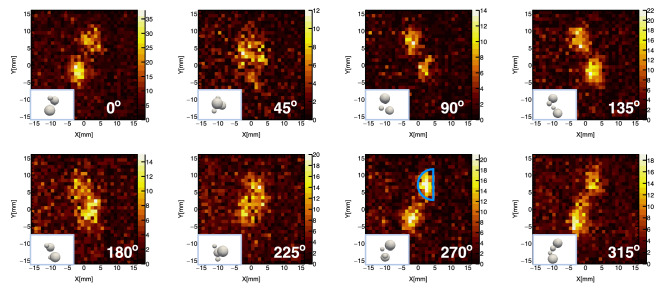
Projection images of the samples taken by the CdTe double-sided strip detector at different rotation angles, along with the actual positioning of the samples (insets). The energy regions of 12–16 keV and 72–78 keV were used to extract all signals of $\upmu$C $L_{\alpha }$ and $\upmu$C $K_{\alpha }$.

The X-ray projection image was obtained based on the orthogonal configuration of the CdTe-DSD electrodes. Since the cathode and anode sides correspond to the x- and y-axes of 2D coordinate geometry, respectively, the intersection point of the signal strips on each side was utilized to reflect the hit position of the corresponding incident photon on the detector. Only the events within the energy ranges of 12–16 and 72–78 keV, respectively corresponding to the $\upmu$C $L_{\alpha }$ and $\upmu$C $K_{\alpha }$ lines, were used to acquire the projection images of the samples for eliminating the background ($\upmu$Al $L_{\alpha }$, 65.9 keV). Figure 3 displays the resulting projection images with a blue frame, which shows the expected signal distribution from the larger sample at the rotation angle of 270$^\circ$. The spherical shapes of the samples and their signal distribution according to the rotational movement could be recognized. It demonstrated that the distribution of emitted muonic X-rays in the samples was successfully revealed by the CdTe-DSD. To further quantify the accuracy of the projection images, the three following aspects were evaluated: the stopping depth of the negative muons in the samples; enlargement effect of the pinhole collimator; size of the projection images and spatial resolution.

First, we estimated via Monte Carlo simulations the stopping depth of a negative muon having a momentum of 30 $\mathrm {MeV}/c$ in a PP matrix. The result was 1.07 mm, with a standard deviation of 0.23 mm. The value suggests a half-round shape of the projection image on the CdTe-DSD rather than a spherical one; this resulted from most of the muons being stopped at the sample surface. Second, the image enlargement effect by the pinhole collimator was investigated based on its distance from the detector ($d_{1}=$ 74 mm) and the samples ($d_{2}=$ 109 mm). According to this effect, we estimated that each projection image was enlarged by a scale factor of $d_{1}/d_{2}$. Hence, when the sample diameter was 12.7 mm (Large-1 and Large-2), the calculated diameter of the corresponding half-round projection was 8.6 mm. This means that the size of the projection image of the larger sample (Large-1 or Large-2) on the y-axis and the x-axis was expected to be 8.6 and 4.3 mm, respectively. Finally, the geometry spatial resolution ($R_{g}$) associated with the pinhole collimated system was estimated as follows^19^:

1 $$\begin{aligned} R_{g} = \sqrt{\left( \frac{d_{1}+d_{2}}{d_{1}}\times r\right) ^{2} + \left( R_{i}\times \frac{d_{2}}{d_{1}}\right) ^{2}}, \end{aligned}$$

where *r* is the diameter of the pinhole collimator (3 mm), and $R_{i}$ is the CdTe-DSD resolution of 250 $\upmu$m. The $R_{g}$ was calculated to be 7.4 mm, which primarily depends on the collimator’s contribution to spatial resolution. Because the partial-volume effect^20^ arises when the sample size is comparable to the spatial resolution of the imaging system, the intensity of the Small-1 and Small-2 with a diameter of 6.35 mm were decreased. Since the projection image from the larger samples at the rotation angle of 270$^\circ$ provided a sufficiently high amount of data, it was selected to investigate its size via the fitting with a Gaussian function. The resulting full width at half maximum (FWHM) values of 3.8 and 7.5 mm revealed the projection image size in the x and y directions, respectively. By considering that the FWHM criterion might give an underestimation of the actual diameter of the samples, these FWHM values are in good agreement with the expected sizes.

### Reconstruction of 3D phantom

**Figure 4:**
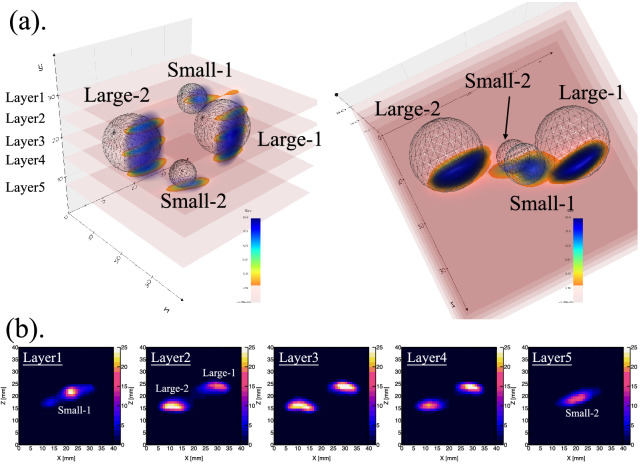
(**a**) Reconstructed three-dimensional (3D) phantom. (**b**) Tomographic images at four different layers perpendicular to the y-axis.

**Table 1:** The resulting mean and FWHM values of the reconstructed balls in the three-dimensional phantom along the x-, y-, and z-axes.

Name	x direction (mm)		y direction (mm)		z direction (mm)	
	Mean	FWHM	Mean	FWHM	Mean	FWHM
Large-1	29.38	7.98	20.98	10.52	23.80	3.29
Large-2	12.01	8.54	19.20	12.29	16.07	3.13
Small-1	22.31	6.61	9.97	4.91	21.72	3.96
Small-2	20.85	8.71	30.97	5.31	19.18	4.12

A reliable method to estimate the 3D distribution of the samples from the projection images was investigated. MLEM, a statistical iterative reconstruction algorithm, was used widely in medical image reconstruction, enabling a more accurate acquisition for the pinhole imaging system than with other mathematical inversion algorithms^21^. For a maximum likelihood estimation of the 3D phantom with the MLEM, we used a set of 16 rotation-based projection images taken by the CdTe-DSD. The algorithm was run until convergence in a gradually optimized iteration number of 50^22^. Figure 4a illustrates the resulting 3D phantom in a 3D Cartesian coordinate system. Also, the 3D phantom was visualized in Supplementary Video. According to the simulated stopping depth of the negative muons, the appearance of the 3D phantom for an individual sample should be close to a circle disk. The tomographic images of the 3D phantom (Fig. 4b) were extracted using dynamic scanning along the y-axis and fitted with the Gaussian smoothing kernels to find the mean and FWHM values. The mean and FWHM values were required to indicate the position and size of 3D phantom, respectively. Table 1 lists the resulting mean and FWHM values along the x-, y-, and z-axes. The small FWHM values for z direction demonstrated the simulation result, which presented that the muons stopped near the sample surface. In addition, because muonic X-rays were emitted from the several micrometers depth of the samples, the sizes of the 3D phantoms of the samples, associated to FWHM in x and y directions, were expected to be smaller than their actual diameter. Yamamoto et al.^23^ reported that the muon beam distance in x direction was shorter than the one in y direction in J-PARC; thus, the 3D phantom sizes of Large-1 and Large-2 along the x-axis were expected to be further smaller due to the asymmetry profile of the muon beam direction. The 3D phantoms of Small-1 and Small-2 were also successfully reconstructed, even the boundaries were strongly affected by the surrounding $\upmu$Al background due to their lower intensity in the projection images. The mean values shown in the Table 1 define the relative position of the 3D phantoms. A quantitative comparison between the mean value of the 3D phantoms in each direction and the actual position of the samples was investigated. Overall, we achieved a reasonably good reconstruction of the 3D phantom based on MLEM algorithm using muonic X-rays. We reported a feasibility study of a non-destructive 3D imaging method based on muonic X-rays for light elements in the bulk samples. To utilize muon beams for the analysis of precious samples that do not allow rotational movements, an advanced detection system comprising several CdTe-DSDs installed at different angles will be developed in the future to acquire all the projection images at the same time.

## Conclusions

This study demonstrates that a non-destructive 3D elemental analysis based on muonic X-rays is feasible for the bulk samples. We prepared four spherical PP balls as the sample for the volumetric reconstruction of a 3D phantom by using negative muons and utilized a CdTe-DSD for data acquisition. The data analysis followed three steps: spectral analysis, projection image acquirement, and 3D phantom reconstruction. In the spectral analysis step, $\upmu$C and $\upmu$Al signals from the samples and the Al columnar stand were respectively observed; in particular, the energy spectrum of the samples showed five peaks at 13.8, 18.7, 74.9, 89.2, and 94.2 keV that were assigned to $\upmu$C $L_{\alpha }$, $\upmu$C $L_{\beta }$, $\upmu$C $K_{\alpha }$, $\upmu$C $K_{\beta }$, and $\upmu$C $K_{\gamma }$, respectively. In the next step, the projection images obtained using the $\upmu$C $L_{\alpha }$ and $\upmu$C $K_{\alpha }$ signals accurately visualized the stopping depths of the incident muons and the revealed shape of the larger samples, which have a diameter of 12.7 mm. Moreover, the visual comparison of the projection images taken at different angles confirmed the rotational movement of the samples. However, the accuracy of the projection images for the smaller samples (Small-1 and Small-2, diameter of 6.35 mm) was strongly affected by the $\upmu$Al background due to its low intensity. In the final step, the inverse problem of 3D phantom reconstruction with the set of rotation-based projection images was dealt with by using the MLEM algorithm. After implementing MLEM algorithm, the 3D phantom was successfully reconstructed, revealing the sample structure and the stopping depth of the muons. The agreement of the 3D phantom was assessed via quantitative comparison with the fitted mean values and actual position. Although the projection images of Small-1 and Small-2 were adversely affected by the $\upmu$Al background, we have succeeded to reconstruct their 3D phantoms using the MLEM algorithm. Our results demonstrate the feasibility of a non-destructive elemental analysis for 3D imaging based on muonic X-ray measurements by a CdTe-DSD. The proposed 3D visualization method can provide an important improvement for elemental analysis in various fields.

## Materials and methods

### Muon experiment

To investigate the feasibility of a non-destructive 3D elemental analysis based on muonic X-ray and a CdTe-DSD, we performed an experiment at the D2 muon beamline of the Muon Science Establishment (MUSE) in J-PARC. The MUSE is an experimental muon facility with the highest-intensity pulsed negative muon beam in the world^24^. The proton beam is accelerated with a power of 1 MW, which corresponds to a beam energy of 3 GeV^25^. The high-intensity negative muon beam is obtained by irradiating the muon production target with the designed proton beam^26^. The momentum of the generated muon beam can be adjusted in the range of 5–120 $\mathrm {MeV}/c$ by a superconducting solenoid magnet installed in the D2 beamline^27^. In our experiment, the muon beam momentum was set at 30 $\mathrm {MeV}/c$ with a 25-Hz operation based on the intensity of the generated muon beam and the expected stopping depth of the negative muons.

### CdTe-DSD

The CdTe-DSD was developed as an imaging detector for hard X-ray and $\gamma$-ray measurements. It has separate readout electronics orthogonally placed on the anode and cathode sides; each electronic system utilizes 128 strip electrodes with a strip pitch of 250 $\upmu$m and the gap width of 50 $\upmu$m. Its sensitivity area and thickness are 32 mm $\times$ 32 mm and 0.75 mm, respectively. The orthogonal configuration corresponds to the 2D coordinate geometry providing information about the interaction position of each incident photon on the detector. According to Hagino et al.^28^, it has a position resolution of a few hundred $\upmu m$. The CdTe-DSD covers an energy range of 5–200 keV in air and has a detection efficiency of 90% at 60 keV^14^. When hard X-rays or $\gamma$-rays deposit their energy in a CdTe semiconductor, free charge carriers, that is, electron-hole pairs, are generated. The electrons and holes are collected by the electronics on the anode and cathode sides, respectively; then, each electrode provides charge signals amplified by the dedicated analog application-specific integrated circuits^29^. The amount of charge deposited in each electrode corresponds to the energy of an incident photon. During the electron and hole transport in the CdTe semiconductor, the charge carriers release their energy to the adjacent strip electrodes. Therefore, this charge splitting property is taken into account for the energy calculation. The energy deposits on a signal strip electrode on each side, $E_{\mathrm {anode}}$ and $E_{\mathrm {cathode}}$, is calculated by summing the energies of its adjacent strip electrodes. To minimize electrical noises such as leakage current, which increases exponentially along with the temperature, the detector was cooled down at $-20 ^\circ$C by a Peltier cooler during the muon irradiation experiment, and a bias voltage of 200 V was applied. In this typical operating condition, we first investigated the CdTe-DSD performance by using an $^{241}$ Am radiation source. A high energy resolution with a FWHM of 1.6 keV at 60 keV was achieved.

In our muon experiment at J-PARC, events with multiple incident photons accounted for 67.2% of the overall measured data. This phenomenon presented a serious analytical challenge, possibly causing the misidentification of the real position of the incident photons. For instance, when it is irradiated by two photons both within its timing window of the CdTe-DSD, two strip electrodes of each anode and cathode sides provide the signals in a signal event ($\mathrm {nSignal}_{\mathrm {anode}} = 2$ and $\mathrm {nSignal}_{\mathrm {cathode}} = 2$). As a result, four intersections of the strip electrodes on the two sides ($\mathrm {nSignal}_{\mathrm {anode}}\times \mathrm {nSignal}_{\mathrm {cathode}} = 4$) are identified as four candidates of the interaction positions of two incident photons. Since the energy deposited on the two sides should be close when the signals come from the same photon, all the intersection point candidates for an event were inspected based on the energy difference between the two sides ($\Delta E=\mid E_{\mathrm {anode}}-E_{\mathrm {cathode}}\mid$). After the charge collections of both sides were confirmed with a certain margin, the candidates with $\Delta E < 5~\mathrm {keV}$ were identified as the incident photons.

### MLEM

**Figure 5:**
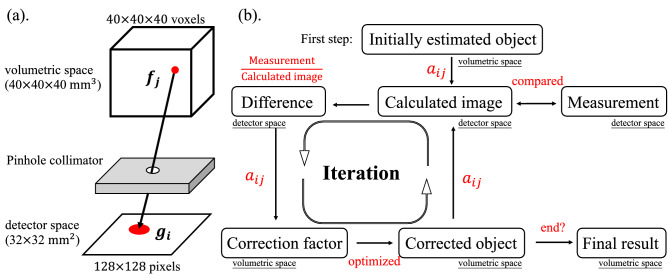
(**a**) Mathematical description of the geometry for the experiment with $^{241}$ Am. (**b**) Maximum likelihood expectation maximization implementation.

The projection images of the samples at different rotation angles were taken by an imaging system consisting of a CdTe-DSD and a 3 mm pinhole collimator, which was designed to ensure a field of view of $50^\circ$. The pinhole collimator, made of tungsten with a thickness of 8 mm, provides the transmission of 250 keV X-ray approximates to $10^{-3}$^14^. The acquired projection images were utilized to obtain a 3D phantom of the samples. We tested two reconstruction algorithms, Filtered BackProjection (FBP)^30^ and MLEM, for this analysis. Although the FBP one requires a shorter computation time to reconstruct a 3D phantom, it is affected by negative artifacts that degraded the imaging performance^31^. To deal with this problem, the MLEM algorithm has been developed based on the CT imaging theory^32^. Since the MLEM algorithm provides more credible results by considering the noise associated with each measurement, it was adopted instead of the FBP in this study for a high-quality 3D phantom reconstruction.

According to the basic principle of MLEM, the measurement of the detector response from the samples in the volumetric space was predicted in a mathematical description of the geometry, as shown in Fig. 5a. We used 40 $\times$ 40 $\times$ 40 voxels with a size of 1 mm and 128 $\times$ 128 pixels with a size of 0.25 mm to describe the volumetric and detector space, respectively, and designed a matrix as a system response between each voxel and pixel. Let $g_i$ and $f_j$ be the vectors for a pixel *i* and a voxel *j*, respectively; the geometry system response can be defined as a matrix ($a_{ij}$). Then, a forward projection can be expressed as:

2 $$\begin{aligned} g_{i} = \sum _{j=1}^{n_{\mathrm {voxels}}} a_{ij}f_{j}. \end{aligned}$$

This equation indicates that the contribution of a planar image in the detector space ($g_{i}$) was impacted by all the emitted sources in the volumetric space with the designed matrix ($\sum _{j=1}^{n_{\mathrm {voxels}}} a_{ij}f_{j}$). According to Shepp and Vardi^33^, the equation is leaded to be an iterative scheme as follows:

3 $$\begin{aligned} f_{j}^{(n+1)} = \frac{f_j^{(n)}}{\sum \nolimits _{i=1}^{n_{\mathrm {voxels}}} a_{ij}} \sum \limits _{i=1}^{n_{\mathrm {voxels}}}\frac{g_{i}}{\sum \nolimits _{j=1}^{n_{\mathrm {pixels}}}a_{ij}f_j^{(n)}}a_{ij} , \end{aligned}$$

where *n* is the $n^{\mathrm {th}}$ iteration of MLEM. In this work, to obtain a matrix ($a_{ij}$) with the dimension of $N_{\mathrm {voxels}}\times N_{\mathrm {pixels}}$, which is associated with the production of dimension number of the voxels and pixels, we placed an $^{241}$Am radioisotope source ($\Phi =$1 mm) at 125 locations in the volumetric space to acquire the corresponding planar images in the detector space. Figure 5a illustrates the experimental setup with $^{241}$ Am. Figure 5b, which is a brief description for MLEM implementation of this analysis, shows how an initial guess object for the 3D phantom of the samples was first designed and, then, the corresponding planar image was calculated with the system matrix $a_{ij}$ by using Eq. (2). The computed planar image was compared with the one acquired from the CdTe-DSD to find the relative difference (“Difference”) and it was backprojected to the volumetric space as an object error map (“Correction factor”) to optimize the initial guess. The estimation of the 3D phantom (“Corrected object”) was improved by repeating this scheme until convergence; finally, a credible solution (“Final result”) could be obtained with a small degree of noise effect.

The MLEM applicability was investigated using a point radioisotope source with a diameter of 1 mm. The planar images observed by the CdTe-DSD through the 3-mm pinhole collimator were used to reconstruct the 3D phantom of the point source. The resulting FWHM of the 3D phantom was 1.17 mm. Thus, the difference between the obtained FWHM value and actual diameter of the point source was 0.17 mm. This result demonstrates the successful 3D phantom reconstruction by using the MLEM algorithm. This MLEM-based method was also applied in the muon experiment to reconstruct the 3D phantom of the samples by using muonic X-rays.

## Supplementary Information

Supplementary Video 1.
